# Metallomics Analysis for Assessment of Toxic Metal Burdens in Infants/Children and Their Mothers: Early Assessment and Intervention Are Essential

**DOI:** 10.3390/biom11010006

**Published:** 2020-12-23

**Authors:** Hiroshi Yasuda, Toyoharu Tsutsui, Katsuhiko Suzuki

**Affiliations:** 1 La Belle Vie Research Laboratory, Tokyo 103-0006, Japan; Tsutsui@lbv.co.jp; 2 Faculty of Sport Sciences, Waseda University, Tokorozawa 359-1192, Japan

**Keywords:** metallomics analysis, toxic metal burdens, zinc deficiency, metal imbalance, metal-metal correlations, Infantile time window, child/mother relationship, early assessment and intervention

## Abstract

Accumulation of toxic metals in infants/children is of serious concern worldwide, from the viewpoint of their harmful effects on the normal growth and development. This metallomics study investigates the extent of toxic metal burdens in infants/children and the relationship to those in their mothers for 77 child/mother pair subjects. For mercury, its geometric mean concentration in infants/children was of similar level to that in their mothers, and a high-significant close correlation was observed between infants/children and their mothers (β = 0.758, r = 0.539, *p* < 0.0001). A significant but less intimate mother/child relationship was observed for arsenic (β = 0.301, r = 0.433), lead (β = 0.444, r = 0.471) and aluminum (β = 0.379, r = 0.451). Remarkably, the burden levels of lead, cadmium and aluminum in infants/children were approximately three times higher than those in their mothers (*p* < 0.0001), and the burden levels in some individuals were several tens of times higher than in the mothers. In contrast, some essential metal levels such as zinc, magnesium and calcium in infants/children were significantly lower than those in their mothers, and 29 individuals (37.7%) in the child subjects were estimated to be zinc-deficient. In addition, significant inverse correlations were observed between zinc and lead (r = −0.267, *p* = 0.019), and magnesium and arsenic (r = −0.514, *p* < 0.0001). These findings suggest that these toxic metal burdens and essential metal deficiencies in infants/children are of serious concern for their neurodevelopment, indicating that the early assessment and intervention are crucial. It is expected that larger epidemiological and intervention studies will provide a reasonable and essential pathway for intervention of neurodevelopment disorders.

## 1. Introduction

Accumulation of toxic metals in infants and children is of serious concern worldwide, from the viewpoint of their harmful effects on normal growth and development of infants and children [1,2,3,4,5,6,7,8]. The pathogenic roles of some toxic metals have been interested in various neurodegenerative diseases [9,10], and mercury, lead, aluminum, and cadmium were reported to cause some fractions of neurodevelopmental disabilities [4,5,6,7,8] and retardation of children’s growth [11,12]. In addition, we demonstrated zinc- and magnesium deficiency and toxic metal burdens such as aluminum, cadmium, lead, arsenic and mercury in autistic children [4,5,13,14], particularly in the infantile subjects aged 0–3 years, and statistically significant inverse relationship between zinc and lead, between zinc and cadmium, and between zinc and aluminum concentrations in these children, indicating the presence of “Infantile Time Window” in neurodevelopment disorders and for intervention [4,5,14]. Cusick and Georgirff pointed out the importance of micronutrients including iron and zinc in early childhood before the age of 3 years in brain development [15]. The critical role of zinc deficiency in etiology of autism has been suggested by recent studies that estimated fetal and postnatal exposure profiles of toxic metals and essential minerals [6,7,16], and that investigated AMPA (α-amino-3-hydroxy-5-methyl-4-isoxazolepropionicacid) receptor subunit switch controlled by zinc and Shank in developing neurons [17,18]. Hagmeyer et al. [19] suggested the beneficial effects of zinc supplementation in treatment of autism and Phelan McDermid Syndrome.

This study was undertaken to investigate the incidence and extent of accumulation of toxic metals in infants/children and to examine whether their toxic metal burdens are affected by those in their mothers. In addition, the concentrations of 13-kind essential minerals in the same subjects were determined simultaneously and the possible relationship between toxic metal burden and deficiency in some essential metals was examined.

## 2. Materials and Methods

### 2.1. Study Subjects

In collaboration with the Food Safety Citizen’s Watch, after obtaining informed consent, 77 pairs of Japanese infants and children (28 males and 49 females; 0–11 year-old) and their mothers were recruited, and their scalp hair samples were collected and used for this study. A hair sample, a kind of cellular body, is widely used as a noninvasive, stable and useful bio-specimen for assessment of environmental exposure of toxic metals, for evaluation of nutritional status and in forensic science [1,3,4,5,8].

The research protocol for examining exposure to main toxic metals, mercury (Hg), arsenic (As), cadmium (Cd), lead (Pb) and aluminum (Al) and their effects in human health and diseases was approved by the ethical committee of the La Belle Vie research laboratory.

### 2.2. Sampling and Metal Analysis

Hair sampling was conducted by cutting hair as close as possible to the scalp of the occipital area. Scalp hair samples were mailed from the child/mother pair subjects to the laboratory.

A 75 mg hair sample was weighed into a 50 mL plastic tube and washed with acetone and then with a 0.01% Triton solution, in accordance with the procedures recommended by the Hair Analysis Standardization Board, as previously described [3,4,5]. The washed hair sample was mixed with 10 mL 6.25% tetramethylammonium hydroxide (TMAH, Tama Chemical, Kawasaki, Japan) and 50 μL 0.1% gold solution (SPEX Certi Prep, Metuchen, NJ, USA), and then dissolved at 75 °C with shaking for 2 h. After cooling the solution to room temperature, an internal standard solution was added. After adjusting its volume gravimetrically, the obtained solution was used for metal analysis. The metal concentrations were determined with inductively coupled plasma mass spectrometry (ICP-MS; 7500ce, Agilent Technologies, Santa Clara, CA, USA) by the internal standard method [3,4,5,13,14] and expressed as ng/g hair (ppb). The reference geometric mean values for each trace element were obtained from the data for 436 male healthy subjects aged 21–40 year-old, as previously reported [4,5,13,20], and used for the calculation of relative concentrations to examine metallome profiles of several pairs of infant/child and mother. All of the data collected were held securely in such a form as to ensure anonymity.

### 2.3. Statistical Analysis

Because scalp hair mineral concentration follows a log-normal distribution, the log of each metal concentration and the geometric rather than arithmetic mean were used to represent the hair metal concentration. Statistical differences between geometric mean concentration of metals in infants/children and in their mothers were analyzed using Welch’s t test. Since there were no significant gender differences in the concentrations of 5-kind toxic metals and 13-kind essential minerals between male and female infants/children (data not shown), the pooled data in a total 77 child subjects was compared with that in their mothers. The relationship of metal concentration between infants/children and their mothers was examined using Pearson’s correlation coefficient. The correlation of some metal-metal pairs in infants/children and in mothers was also analyzed by the same method.

## 3. Results

**Figure 1:**
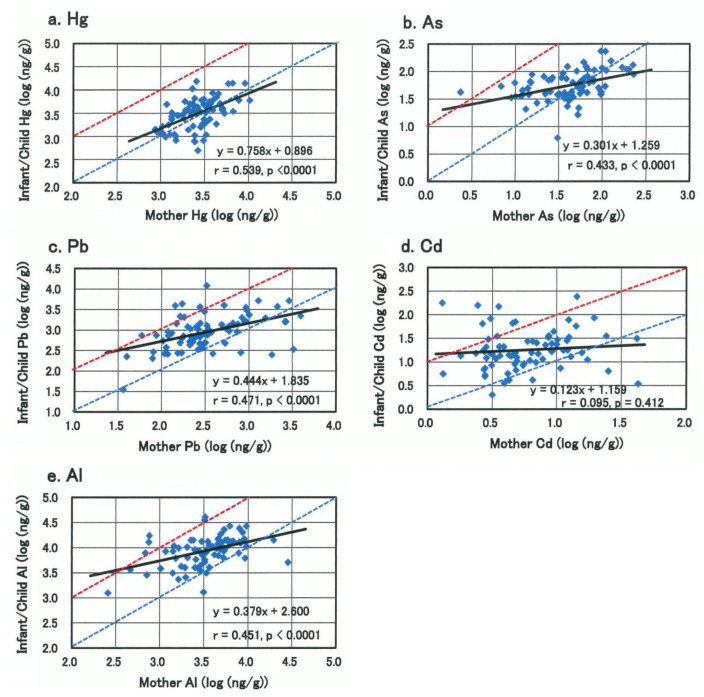
Relationship of toxic metal concentrations in scalp hairs between infants/children and their mothers. (**a**) Mercury, (**b**) Arsenic, (**c**) Lead, (**d**) Cadmium, (**e**) Aluminum. Dotted blue line and red line represent the toxic metal concentrations in infants/children equal to and 10 times higher than those in their mothers, respectively.

Metallomics analysis of 26-kind of trace bio-elements including toxic metals in scalp hair samples of 77 pair subjects of Japanese infants/children (28 males and 49 females; 0–11 year-old; mean age: 5.2 years old) and their mothers (mean age: 38.4 years old) were investigated in this study. Geometric mean concentration of mercury in infants/children was not statistically different from that in their mothers, and a high-significant close correlation (β = 0.758, r = 0.539, *p* < 0.0001) was observed between mothers and infants/children (Figure 1a), suggesting equivalent exposure to mercury in infants/children and mothers. A significant but less intimate mother/child relationship was observed for arsenic (β = 0.301, r = 0.433), lead (β = 0.444, r = 0.471), and aluminum (β = 0.379, r = 0.451) (Figure 1b–e), and these mean burden levels in infants/children were significantly higher than those in their mothers (Table 1). In particular, for lead, cadmium and aluminum, these mean burden levels in infants/children were approximately three times higher than those in their mothers, and the burden levels in some individuals exhibited several tens of times higher than those in their mothers. Namely the maximum values for lead, cadmium, and aluminum in infants/children were 11,930 (1-year-old girl), 241 (2-year-old boy) and 40,740 ng/g (2-year-old boy), which were 35, 41, and 12 times higher than mean levels in the mothers, respectively (Table 1).

**Figure 2:**
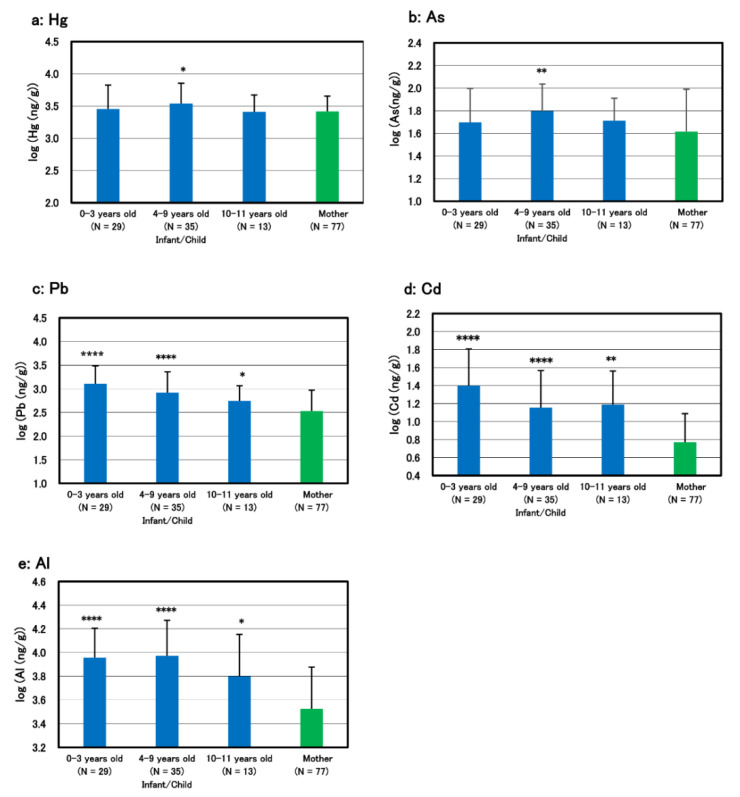
Geometric mean toxic metal concentrations in infants/children grouped by age and their mothers. (**a**) Mercury, (**b**) Arsenic, (**c**) Lead, (**d**) Cadmium, (**e**) Aluminum. Infants/children were grouped by age; 0–3, 4–9, and 10–11 year-old, and the geometric mean concentrations of toxic metals in each group were compared to those in their mothers. Statistically significant difference in the mean value in each age-group compared to their mother group is shown with asterisk marks: * *p* < 0.05, ** *p* < 0.01, **** *p* < 0.0001.

Infants/children were divided into three groups by age; 0–3, 4–9, and 10–11 year-old, and mean concentrations of the five toxic metals in each group were calculated and compared to their mothers (Figure 2). The mean burden levels of lead, cadmium, and aluminum in infant group of 0–3 year-olds were highest, high-significantly different from their mothers (*p* < 0.0001) and declined with age (Figure 2c–e), while such an age-dependency was not observed in those of mercury and arsenic (Figure 2a,b).

Among essential minerals, the levels of some elements, such as chromium, molybdenum, manganese, iron, and iodine in infants/children were significantly nearly two times higher than those in their mothers as well as for the above toxic metals. In contrast, the limited three elements of zinc, magnesium and calcium in infants/children were significantly lower than those in their mothers (*p* < 0.0001) (Table 2), and 29, 9 and 8 individuals (37.7, 11.7 and 10.4 %) in the infants/children group were estimated to be zinc-, magnesium- and calcium-deficient, respectively. There were a few or no individuals with deficiency in iron, copper, manganese, chromium, and molybdenum.

The mutual relationship of these toxic metals and essential minerals were examined by multiple regression analysis, and the correlation coefficients between some metal-metal combinations are shown in Table 3. In infants/children, statistically significant positive correlations were observed between cadmium and lead (r = 0.616, *p* < 0.0001), aluminum and lead (r = 0.347, *p* = 0.002), and aluminum and iron (r = 0.601, *p* < 0.0001). In their mothers, similar positive relationships were observed in each metal-metal combination (r = 0.438, *p* < 0.0001; r = 0.273, *p* = 0.016; r = 0.426, *p* = 0.0001, respectively). About iron element, not only these toxic metals but also various metals such as manganese, cobalt, chromium, molybdenum, and nickel so on positively correlate with iron (data not shown), suggesting co-burdens of iron with a variety of heavy metals.

In contrast, statistically significant inverse correlations were observed between zinc and lead (r = −0.267, *p* = 0.019), between magnesium and arsenic (r = −0.514, *p* < 0.0001) and between zinc and molybdenum (r = −0.331, *p* = 0.003) in infants/children, and in mothers between magnesium and arsenic (r = −0.649, *p* < 0.0001). It is emphasized that zinc element is only one heavy essential metal exhibiting inverse relationship between various heavy metals, as previously reported [4,5,20]. Among minerals, the highest positive correlation is observed between calcium and magnesium in both the infants/children and mothers group (r = 0.871 and 0.820, respectively, *p* < 0.0001).

**Figure 3:**
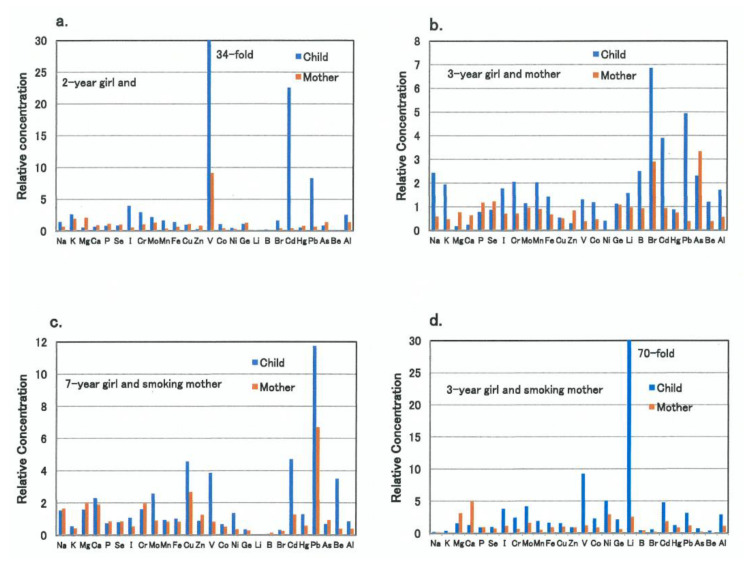
Metallome profiles of four child/mother pairs with some toxic metal burdens and/or essential metal deficiency. Each bar (blue and red) represents the relative concentration of the respective trace element in child’s and the mother’s scalp hair specimens, respectively. The 1.0 of the relative concentration represents the reference control level of each trace element. (**a**) A typical metallome profile of a 2-year-old girl and her mother is shown, exhibiting severe zinc deficiency and simultaneous high burdens with vanadium, cadmium, and lead in the child. (**b**) Another typical metallome profile of a 3-year-old girl and her mother is shown, exhibiting severe magnesium deficiency and simultaneous burdens with lead, cadmium, and bromine in the child. (**c**) A typical metallome profile of a 7-year-old girl and her smoking mother is shown, exhibiting severe lead burden and simultaneous burdens with cadmium, copper, and vanadium in the child. (**d**) Another typical metallome profile of a 3-year-old girl and her smoking mother is shown, exhibiting sever lithium burden and simultaneous burdens with vanadium, nickel, and cadmium in the child.

Representative metallome profiles of four individuals with high burdens of some toxic metals and/or with deficiency in essential metals are shown in Figure 3, together with those of their mothers. The first (Figure 3a) and second (Figure 3b) cases showed a marked zinc deficiency with high burdens of cadmium and lead, while concentrations of these three metals in the mothers were almost normal levels. In the first case, a marked high burden of vanadium was also observed, and its burden level was nearly four times higher than that of her mother. The third and fourth cases, whose mother had a habit of smoking, showed high accumulation of not only cadmium and lead, but also vanadium, copper or lithium than the respective mother, while zinc levels were normal (Figure 3c,d).

## 4. Discussion

The pathogenic roles of some toxic metals such as mercury, lead, aluminum, or cadmium have been interested in various neurodegenerative diseases [1,2,3,8,9,10]. These toxic metals are also known to cause some fraction of neurodevelopmental disabilities [4,5,6,7,14]. Gardner et al. [11] reported that early-life cadmium exposure had the strongest and most consistent inverse association with children’s growth in terms of weight and height. Choi et al. [12] demonstrated that post-birth weight gain was negatively associated with blood levels of lead, and similar negative association for current head circumference with arsenic and lead.

In this study, the geometric mean concentration of mercury in infants/children was of similar level to that in their mothers (Table 1), and statistically high-significant close correlation between mothers and infants/children was observed (Figure 1a), suggesting equivalent exposure to mercury in infants/children and mothers due to their common lifestyles. A significant but less intimate mother/child relationship was observed for arsenic, lead and aluminum (Figure 1b–e), and the proportion of infants/children with higher burdens than mother was larger than that of mercury, and these mean burden levels in infants/children were significantly higher than those in their mothers (Table 1). Remarkably, the mean burden levels of lead, cadmium and aluminum in infants/children were found to be approximately three times higher than those in their mothers, the differences being statistically high-significant (Table 1). The burden levels of these three toxic metals in some infants/children were several tens of times higher than those in mothers (Figure 1c–e). It should be noted that the burden levels of lead, cadmium and aluminum in mothers, whose infants/children showed maximal or sub-maximal levels, were not so high. These findings indicate that these three toxic metals are liable to accumulate in infants/children more than in their mothers.

The burden levels of lead, cadmium, and aluminum in infants of 0–3 year-old were highest and declined with age inversely, while such an inverse age-dependency was not observed in those of mercury and arsenic (Figure 2). These results are coincident with our previous study, suggesting that the three toxic metals of lead, cadmium and aluminum are characteristic liable to accumulate in infants and different from mercury and arsenic which have a character accumulating in adults age-dependently [3]. In addition, statistically significant positive correlations were observed between cadmium and lead, and between aluminum and lead in both infants/children and mothers (Table 3), suggesting that the mechanisms of distribution, detoxification, and excretion of these three toxic metals are different from those of mercury and arsenic. High close correlation between aluminum and iron in infants/children (Table 3) seems to suggest multiple pollutions with some related-metals such as manganese and chromium, as shown in Table 2.

In contrast to toxic metals, the mean concentrations of magnesium, calcium and zinc in infants/children were significantly lower than those in their mothers, and 29 and 9 individuals (37.7 and 11.7%) in the 77 child subjects were estimated to be zinc- and magnesium-deficient, respectively (Table 2). In addition, statistically significant inverse correlations were observed between concentrations of zinc and lead, and between magnesium and arsenic in infants/children (Table 3). A significant inverse correlation between magnesium and arsenic was also observed in their mothers, but not between zinc and lead, suggesting characteristic interaction between zinc and lead in infants/children [4,5,14]. No significant correlation between zinc and cadmium or between zinc and aluminum was observed in this study, but our previous study for larger number of autistic children has revealed high burdens of lead, cadmium and aluminum accompanied by deficiency in zinc and magnesium, and significant relationships between these toxic metal burdens and zinc deficiency [3,4,5,14,20]. It is, therefore, speculated that the relative and absolute deficiency in antagonistic metals such as zinc and magnesium are associated with the accumulation of toxic metals as observed in this study.

In fact, dietary restriction-induced zinc deficiency has been reported to up-regulate intestinal zinc-importer (Zip4) and induce the increase in Zip4 protein located to the plasma membrane of enterocytes [21,22,23,24]. This adaptive response to zinc deficiency is known to induce increasing in the risk of high uptake of toxic metals such as cadmium and lead [25]. Thus, infants with zinc deficiency are liable to be exposed to increased risk of absorbing high amount of toxic metals and retaining them in their body. In addition, maternal cigarette smoking was reported to be associated with lower zinc and higher cadmium and lead concentrations in their neonates [26]. Because of their high distribution property to bone tissue, during pregnancy and lactation, these toxic metals accumulated in the maternal bone matrix are co-transferred with calcium and magnesium to fetal and newborn bodies through increased bone-resorption [27,28].

As shown in Figure 1, toxic metal burden levels in infants appear to be affected profoundly by those in their mothers through placental transfer during pregnancy and breastfeeding after birth, which are affected by foods/drinks, smoking, or passive smoking and other environmental factors. High contents of toxic metals (cadmium, lead and aluminum) in tobacco and high exposure to these toxic metals in smokers and their family members have been reported [29,30,31,32]. Toxic metal burdens in infants are also affected by infant formulas or weaning foods: higher concentration of cadmium in soy-based and cereal-based formulas than cow’s milk formulas and high content of aluminum in infant formulas and vaccine preparations have been reported [33,34,35,36,37].

It is known that breast-fed infants are prone to develop trace element deficiency and that alopecia and dermatitis are attributable to zinc deficiency. Furthermore, “follow-up milk” formulas (nutritional supplement for infants aged over 6-month) used in Japan contain practically iron and calcium fortified but neither zinc nor copper, and the dependence on the follow-up milk formulas as the main source of nutrition is reported to be associated with a high risk of zinc deficiency [38].

Recently, Fiore et al. [8] reported that there was a negative, significant association between hair zinc level and severity of autistic symptoms (defective functional play and creativity and increase of stereotyped behavior), and lead, molybdenum and manganese hair levels were inversely correlated with cognitive level (full intelligence quotient) in autism spectrum disorder (ASD) individuals.

Thus, it is suggested that supplementation of deficient essential metals such as zinc and magnesium in early period of infant/child is expected to ameliorate the body metal imbalances and probably the clinical symptoms related to their deficiencies and toxic metal burdens in the early neurodevelopment period, named “Infantile Time Window” or “First 1000 Days” [4,5,13,14,15,19,39,40].

Arnold et al. [41] reported that mean serum zinc levels were significantly lower in both autism and attention deficit/hyperactivity disorder (ADHD) groups, and that serum zinc level correlated inversely with parent- and teacher-rated inattention in ADHD children. Furthermore, it is reported that zinc supplementation reduces symptoms of hyperactivity, impulsivity, and impaired socialization in ADHD patients [42,43]. Other preliminary human study showed that zinc supplement as an adjunct to methylphenidate has favorable effects in the treatment of ADHD children [44]. The critical role of zinc deficiency in etiology of ASD has been suggested by recent studies showing high uptake of neurotoxin lead and reduced uptake of essential minerals manganese and zinc during specific developmental windows [4,5,6] and disruption of zinc-copper rhythmicity [7] in those children. Furthermore, it has been suggested that zinc signaling through Shank2 and Shank3 controls the AMPA receptor subunit switch in developing neurons, and that zinc deficiency may impair synaptic maturation and circuit formation that underlie ASD etiology [17,18,19]. Recently, Miyake et al. [45] found the association between maternal smoking and DNA hypo-methylation of zinc-dependent Shank2 synaptic scaffolding gene in cord blood, suggesting the epigenetic and unfavorable effect of smoking on neurodevelopment in infants and fetus.

Kozielec et al. [46] reported that in hyperactive children with ADHD, magnesium deficiency was found in the 95% of the subjects and reported that a significant decrease of hyperactivity and increase in hair magnesium contents has been achieved in the group of ADHD children given six months of magnesium supplementation [47]. Mousain-Bosc et al. [48] also reported that 52 hyper-excitable children have low intra-erythrocyte magnesium levels with normal serum magnesium values and that magnesium/vitamin B6 supplementation can restore the erythrocyte magnesium levels to normal and improve their abnormal behaviors. They also reported that 33 children with clinical symptoms of pervasive developmental disorder (PDD) or autism exhibit significantly lower red blood cell magnesium values and that the combination therapy with magnesium/vitamin B6 for six months improved significantly PDD symptoms with concomitant increases in intra-erythrocyte magnesium values [49].

Such an evidence-based nutritional approach supplementing deficient nutrients and detoxifying accumulated toxic metals is of great importance for normal growth and development of infants and children, and probably yields a new pathway into prevention and treatment of neurodevelopment disorders such as ASD, ADHD and also learning disorders [4,5,13,19,50].

The present study was cross-sectional, and detailed information about health conditions, dietary habits and environmental factors of the subjects was lacking. Nevertheless, a careful assessment and monitoring of such infants and children suffered from some essential metal deficiencies and/or toxic metal burdens revealed in this study would be essential, and if necessary, supplementing deficient metals should be considered. Therefore, it is expected that larger epidemiological and intervention studies will clarify the pathogenic relationship between infantile metal imbalances described above and neurodevelopment disorders and thereby provide reasonable and essential procedures for their normal growth and neurodevelopment. In addition, clinical metallomics (or “mineralomics”) analysis with another micronutrient omics “vitaminomics” analysis will be used as an essential tool for personalized precision medicine in the near future.

## 5. Conclusions

In this cross-sectional study of 77 pairs of Japanese infants/children and their mothers, a high-significant close child/mother relationship was demonstrated for mercury burden levels. For the other toxic metals such as lead, cadmium and aluminum, these burden levels in children, particularly in infants, were markedly higher than those in their mothers, with less intimate child/mother relationship. In contrast, the levels of zinc, magnesium, and calcium, which can compete against and antagonize toxic metals, were found to be significantly low in infants/children. High burdens of toxic metals and deficiencies of antagonistic essential metals in infants/children are of serious concern, and early assessment and intervention for such subjects would be helpful for their normal growth and development.

## Figures and Tables

**Table 1:** Mean toxic metal concentrations in scalp hairs of infant/child and their mothers.

	Geometric Mean (ng/g)					
Toxic Metal	Mother	Infant/Child		Maximum	In Infant/Child	
	(N = 77)	(N = 77)		(ng/g)	Ratio	
Hg	2621	3057		15,140	5.8	(F 5)
As	41.2	55.5	*	233	5.7	(M 4)
Pb	338	907	****	11,930	35.3	(F 1)
Cd	5.9	17.9	****	241	40.8	(M 2)
Al	3362	8645	****	40,740	12.1	(M 2)

Statistically significant difference in the mean value in infant/child group compared to their mother group is shown with asterisk marks: * *p* < 0.05, **** *p* < 0.0001. Maximum value for each toxic metal concentration in infant/child and ratio to mother is shown. Alphabet (M, F) and numeral in parentheses represent gender (male, female) and age of the subject who showed maximum value for each metal.

**Table 2:** Mean essential mineral concentrations in scalp hairs of infant/child and their mother.

	Geometric Mean (ng/g)			Deficient Infant/Child	
Essential Mineral	Mother	Infant/Child	Significance	No.	Rate
	(N = 77)	(N = 77)			(%)
Na	16,804	13,307	NS		
K	10,542	17,976	**		
Mg	76,513	27,929	****	9	11.7
Ca	710,404	294,933	****	8	10.4
P	117,833	102,574	NS		
Se	587	577	NS	0	0
I	196	404	***	0	0
Cr	34.9	74.1	****	2	2.6
Mo	33.1	59.1	****	0	0
Mn	108	174	***	0	0
Fe	4514	7922	****	1	1.3
Cu	22,967	19,175	NS	0	0
Zn	135,620	97,139	****	29	37.7

Statistically significant difference in mean concentrations in infants/children group compared to their mother group is shown with asterisk marks: ** *p* < 0.01, *** *p* < 0.001, **** *p* < 0.0001.

**Table 3:** Relationship of metal-metal pair concentrations in infants/children and in their mothers.

	Infant/Child			Mother		
Metal-Metal	(N = 77)			(N = 77)		
Correlation	Coefficient	*p*-Value		Coefficient	*p*-Value	
Cd-Pb	0.616	0.000	****	0.438	0.000	****
Al-Pb	0.347	0.002	**	0.273	0.016	*
Al-Fe	0.601	0.000	****	0.426	0.001	***
Pb-Zn	−0.267	0.019	*	0.139	0.227	
As-Mg	−0.514	0.000	****	−0.649	0.000	****
Zn-Mo	−0.331	0.003	**	−0.183	0.112	
Ca-Mg	0.871	0.000	****	0.820	0.000	****

Statistically significant correlation is shown by p-value and asterisk marks: * *p* < 0.05, ** *p* < 0.01, *** *p* < 0.001, **** *p* < 0.0001.
